# Multi-institutional analysis of independent predictors for burn mortality in the United States

**DOI:** 10.1186/s41038-018-0127-y

**Published:** 2018-08-22

**Authors:** Dmitry Zavlin, Vishwanath Chegireddy, Stefanos Boukovalas, Anna M. Nia, Ludwik K. Branski, Jeffrey D. Friedman, Anthony Echo

**Affiliations:** 1 Institute for Reconstructive Surgery, Houston Methodist Hospital, Weill Cornell Medicine, 6560 Fannin Street, Scurlock Tower, Suite 2200, Houston, TX 77030 USA; 2 Division of Plastic Surgery, The University of Texas Medical Branch, Galveston, TX USA; 3 School of Medicine, The University of Texas Medical Branch, Galveston, TX USA

**Keywords:** Burns, Mortality, Death, Risks, Outcomes

## Abstract

**Background:**

Previous reports individually identified different factors that predict death after burns. The authors employed the multi-center American Burn Association’s (ABA) National Burn Repository (NBR) to elucidate which parameters have the highest negative impact on burn mortality.

**Methods:**

We audited data from the NBR v8.0 for the years 2002–2011 and included 137,061 patients in our study. The cases were stratified into two cohorts based on the primary outcome of death/survival and then evaluated for demographic data, intraoperative details, and their morbidity after admission. A multivariable regression analysis aimed to identify independent risk factors associated with mortality.

**Results:**

A total of 3.3% of patients in this analysis did not survive their burn injuries. Of those, 52.0% expired within 7 days after admission. Patients in the mortality cohort were of older age (*p* < 0.001), more frequently female (*p* < 0.001), and had more pre-existing comorbidities (*p* < 0.001). Total body surface area (TBSA), inhalation injury, hospitalization time, and occurrence of complications were higher compared to survivors (*p* < 0.001). Lack of insurance (odds ratio (OR) = 1.84, confidence interval (CI) 1.38–2.46), diabetes (OR = 1.24, CI 1.01–1.53), any complication (OR = 4.09, CI 3.27–5.12), inhalation injury (OR = 3.84, CI 3.38–4.36), and the need for operative procedures (OR = 2.60, CI 2.20–3.08) were the strongest independent contributors to mortality after burns (*p* < 0.001). Age (OR = 1.07, CI 1.06–1.07) and TBSA (OR = 1.09, CI 1.09–1.09) were significant on a continuous scale (*p* < 0.001) while overall comorbidities were not a statistical risk factor.

**Conclusion:**

Uninsured status, inhalation injury, in-hospital complications, and operative procedures were the strongest mortality predictors after burns. Since most fatal outcomes (52.0%) occur within 7 days after injury, physicians and medical staff need to be aware of these risk factors upon patient admission to a burn center.

## Background

Over the past years, various healthcare registries established enormous databases of information, which include patient characteristics, diagnostic codes, therapeutic management, and complication profiles. The massive volume of medical records allows researchers to investigate questions that otherwise would be impossible to address due to the logistical constraints of individual centers. The concept of burn database development was originally introduced by Dr. Irving Feller from the University of Michigan, who established the National Burn Information Exchange in 1964. With the American Burn Association (ABA) as a spearhead, this program has substantially evolved by creating an electronic burn registry [1]. In 2001, the ABA assumed the responsibility of the national database, which is now known as the National Burn Repository (NBR) [2]. This database has played a vital role in collecting information on patients who receive treatment at burn centers in the United States (US) and provides valuable resources for investigative burn research [3].

Research in the field of burns has been translated to improved survival rates, decreased hospital length of stay, reduced morbidity and mortality rates due to the development of resuscitation protocol, infection control, early wound debridement, early enteral nutrition, respiratory support, and support of hypermetabolic responses. According to federal surveys, there were 486,000 burn injuries reported in the US in 2016 alone that required medical treatment. Of those, a total of 3275 resulted in death [4].

Several studies over the past decade have exploited the enormous information available in the NBR to improve care in burn injuries and reduce mortality rates. Carr et al. have reviewed the NBR from 1998 to 2007 to determine the outcomes in burn patients with inhalation injury complicated by pneumonia [5]. Bedri et al. demonstrated higher mortality rates after burn injuries associated with lower socioeconomic status, female gender, and African-American race [6]. Osler et al. used the database to create a logistic regression model that showed how age, burned surface area, and inhalation injury contributed to mortality. Based on this analysis, they proposed a revised Baux score to improve predictions of mortality after burn injuries [7].

Age, burn size, and inhalation injury have been repeatedly reported as contributors to unfavorable outcomes [5, 6, 8]. However, we intended to expand on these studies and evaluate the importance of other factors as well. The objective of this report is to employ the large multi-institutional NBR database to identify the predictors of mortality after burn injuries by utilizing a detailed regression model controlling for confounders. Furthermore, the time to death is evaluated for those patients with fatal outcomes.

## Methods

### Patients

**Fig. 1:**
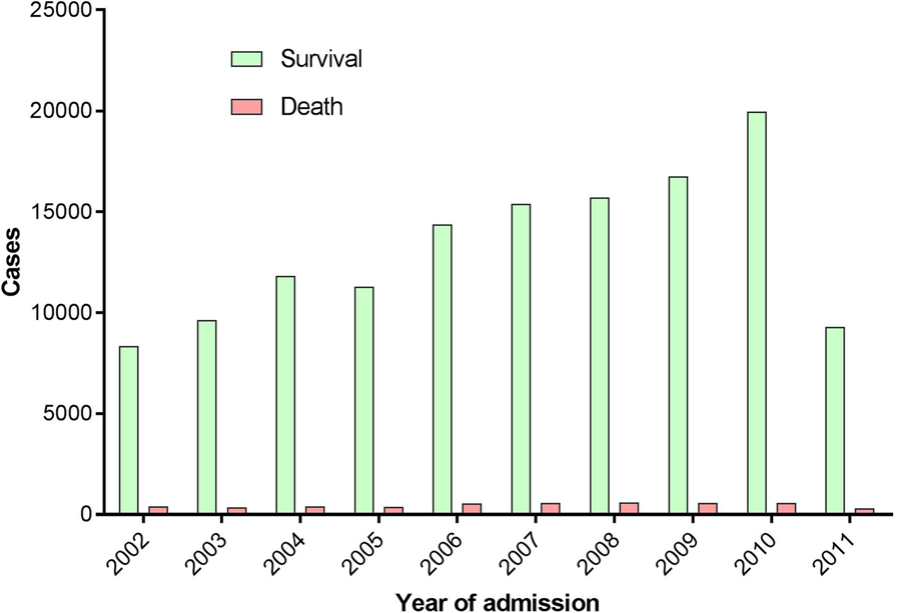
Number of burn cases recorded in the National Burn Respository (NBR) and stratified by year and outcome. As shown, the number of burn cases reported to the NBR from United States (US) burn centers has been increasing annually since 2002. During this period, the proportion of patients passing away from their injuries has been steady at around 3–4%. Unfortunately, for the last year available to the public (2011), only partial NBR data are available

**Fig. 2:**
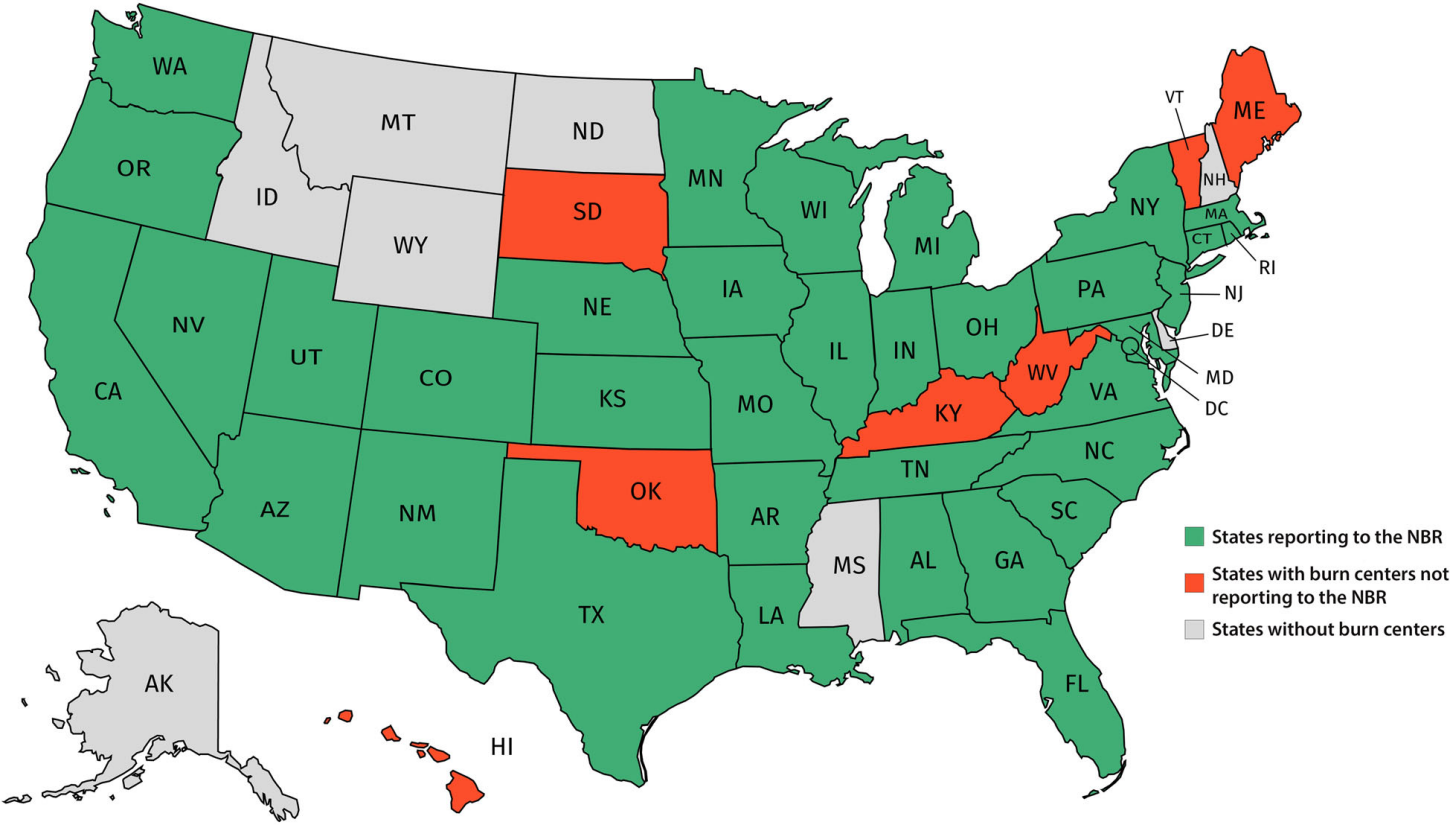
Map of the United States (US) and states participating in the National Burn Respository (NBR). This map shows which US states host American Burn Association (ABA)-approved burn centers that upload data to the NBR (green), do not upload data to the NBR (red), or do not have any burn centers at all (gray)

**Fig. 3:**
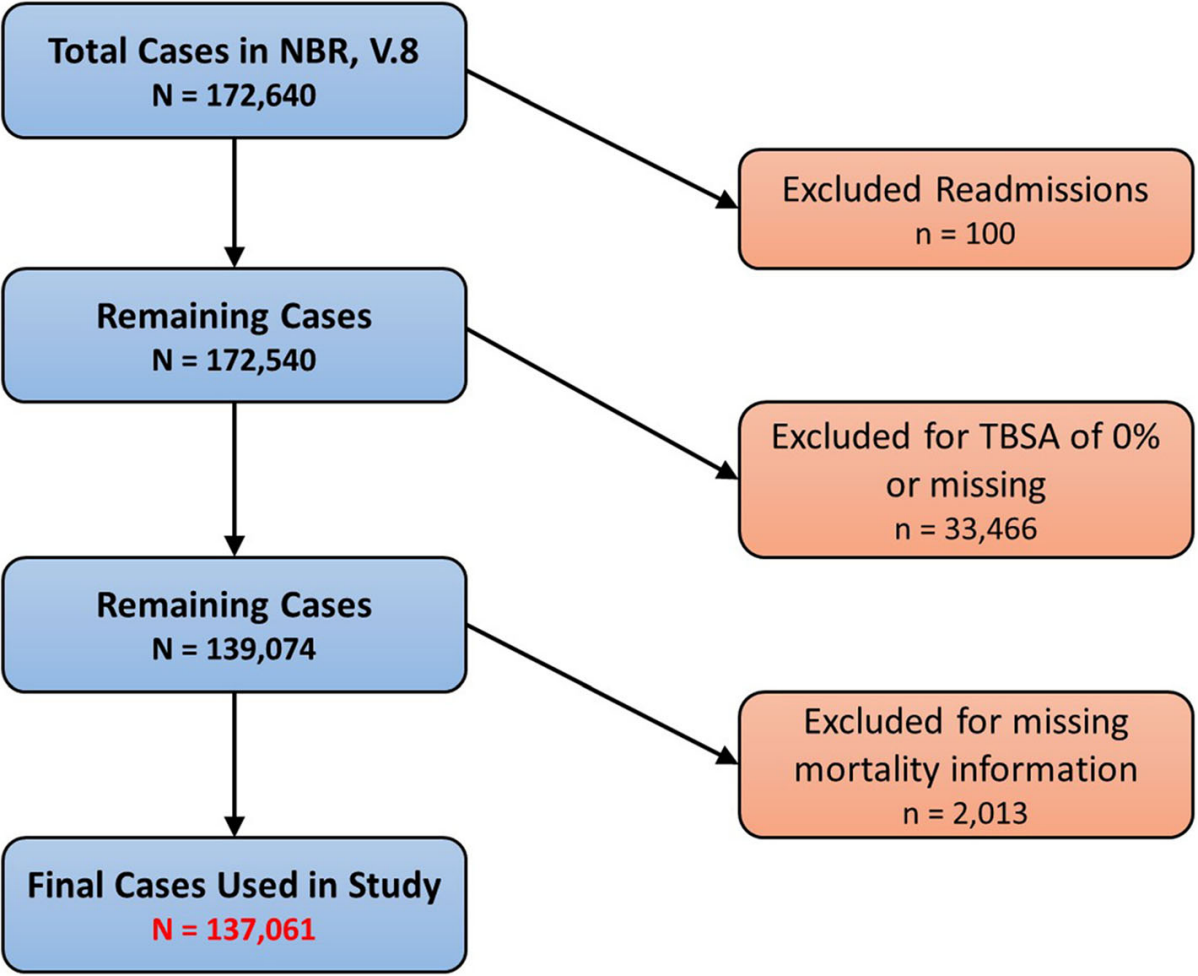
Flowchart depicting case selection and exclusion process. In order to work with accurate data, which was provided by the National Burn Respository (NBR), our group first had to clean up the database and remove certain cases based on the methodology shown in this figure. *TBSA* Total body surface area

Data sources for our study were the eighth and latest version of the NBR dataset provided by the ABA which contained patient information of children as well as adults between 2002 and 2011 (Fig. 1). This repository is updated prospectively and on a regular basis by the participating ABA institutions that are certified burn centers (Fig. 2). Initially, a total of 172,640 cases were extracted from the entire dataset. After removing re-admissions (100), patients with a total body surface area (TBSA) burn of 0% or missing data (33,466), and those with missing mortality information (2013), the remaining 137,061 patients were included in this study (Fig. 3). We created two cohorts based on whether a patient passed away after the burn injury or survived and was discharged from the burn center.

The majority of variables are provided in the primary datafile “Main_v8.” We collected patient demographics, preoperative comorbidities, treatment details, and subsequent outcomes, including morbidity and mortality, and compared between the two cohorts. Demographics included age, gender, race, social factors, and the most prevalent comorbidities. Clinical details entailed variables, such as burn type, partial- and full-thickness burned TBSA, duration of hospitalization, and need for intubation/ventilation or operative procedures. The outcomes were measured by frequency of complications and an evaluation of the cause of death within the study cohort.

Comorbidities and complications are listed in separate datafiles named “Comorbidities_v8” and “Complications_v8,” respectively. In these files, only the presence of variables is documented for each patient.

Finally, a logistic regression analysis aimed to detect independent risk factors associated with death following a burn injury. Written consent from the ABA is available for the use of this database. No institutional review board approval was obtained at our home institution since the datasets are provided in de-identified format.

### Statistics

Various descriptive statistics were created for both patient cohorts meeting the inclusion criteria. The univariate analysis compared the two cohorts’ demographics, clinical details, and final outcomes. The statistical level of significance was set at 5% (*p* < 0.05) for all calculations using the unpaired sample *t* test for continuous and the chi-square test for categorical variables. Cases with missing variables were omitted from the calculations and their number shown separately.

A number of variables were gathered for a multivariable logistical regression analysis in order to control for confounders and to quantify the influence of various factors on mortality rates. Odds ratios (OR) and 95% confidence intervals (CI) of independent variables, such as age, gender, race, uninsured status, comorbidities, complications, inhalation injury, TBSA, and operative procedures, were calculated in their statistical association to death as a dichotomous dependent outcome. By adding multiple independent variables into one single regression model, the influence of each risk is controlled with regard to the other variables if any confounders should exist [9].

The statistical aspects of this study were conducted using SPSS version 24.0 (IBM Corp., Armonk, NY).

## Results

Of all patients included in this study, 132,531 (96.7%) survived their burn injury while 4530 (3.3%) did not. On average, the mortality cohort was much older (56.5 vs. 30.5 years, *p* < 0.001) and had a higher proportion of female patients (35.3% vs. 30.7%, *p* < 0.001) although the male gender was overall predominant. The deceased subgroup had a much lower proportion of children under the age of 18 years (6.1% vs. 32.9%, *p* < 0.001). Caucasians and African-Americans were slightly more present in our cohort of patients who did not survive their burn injury. There was no difference in the number of uninsured people between both groups. However, patients who passed away over the course of their burn injury were more likely to be smokers (5.8% vs. 5.0%, *p* = 0.022), illicit drug users (2.9% vs. 2.2%, *p* = 0.005), and suffering from alcoholism (5.0% vs. 2.9%, *p* < 0.001). In addition, these deceased patients had higher ratios of any kind of comorbidity documented by the NBR compared to the control group of survivors (26.6% vs. 14.5%, *p* < 0.001). These discrepancies remained highly significant when analyzing specific diseases separately (Table 1).

**Table 1:** Demographic variables of the burn patients

Variable	Survival, *n* = 132,531		Death, *n* = 4530		*P* value
	Missing	Value	Missing	Value	
Age, years (mean ± SD)	30,542	30.5 ± 22.4	704	56.5 ± 22.5	< 0.001*
Children < 18 years, n (%)		33,601 (32.9)		233 (6.1)	< 0.001*
Gender, n (%)	0		0		< 0.001*
Male		91,814 (69.3)		2933 (64.7)	
Female		40,717 (30.7)		1597 (35.3)	
Race, n (%)	5564		197		< 0.001*
Caucasian		74,403 (58.6)		2835 (65.4)	
African-American		24,024 (18.9)		878 (20.3)	
Hispanic		19,770 (15.6)		388 (9.0)	
Other		8770 (6.9)		232 (5.3)	
Alcohol, n (%)	N/A	3792 (2.9)	N/A	226 (5.0)	< 0.001*
Illicit drug use, n (%)	N/A	2959 (2.2)	N/A	130 (2.9)	0.005*
Smoker, n (%)	N/A	6685 (5.0)	N/A	263 (5.8)	0.022*
Uninsured, n (%)	14,057	17,426 (14.7)	538	590 (14.8)	0.903
Comorbidities, any^a^, n (%)	N/A	19,281 (14.5)	N/A	1206 (26.6)	< 0.001*
CHF		871 (0.7)		147 (3.2)	< 0.001*
Diabetes		3901 (2.9)		291 (6.4)	< 0.001*
Hypertension		8850 (6.7)		631 (13.9)	< 0.001*
Psychiatric illness		4512 (3.4)		227 (5.0)	< 0.001*
Obesity		1405 (1.1)		87 (1.9)	< 0.001*
Respiratory disease		5464 (4.1)		291 (6.4)	< 0.001*

*Significant with *p* < 0.05

^a^Alcoholism, bleeding disorder, chemotherapy, congenital anomalies, CHF, connective tissue disease, current smoker, dialysis, CVA, diabetes mellitus, cancer, drug abuse, esophageal varices, dependent health status, history of angina/MI, hypertension, immunodeficiency, neurological impairment, obesity, other, prematurity, psychiatric illness, respiratory disease, steroid use, and transplant of organ(s)

*CHF* congestive heart failure, *CVA* cerebrovascular accident, *MI *myocardial infarction, *N/A* not available, *SD* standard deviation

Those patients who died after their injury were more likely to have experienced fire or flame burns (47.6%) in relation to those who survived (24.5%, *p* < 0.001). Their TBSA for both split- and full-thickness burns were much higher (both *p* < 0.001) and an indicator for more severe injuries. In addition, patients with lethal burns had significantly more inhalation injuries (46.1% vs. 5.7%), which required more intubations (80.9% vs. 9.8%), and were subsequently ventilated and in an intensive care unit for an extensively prolonged time (all *p* < 0.001, Table 2). With a longer mean hospitalization time (18.0 vs. 9.4 days), higher number of operations (2.4 vs. 1.0), and a greater number of total surgical procedures (4.0 vs. 1.7), the care for patients who did not survive generated much higher costs, $264,000 vs. $79,000 (all *p* < 0.001, Table 2).

**Table 2:** Clinical details of the burn patients

Variable	Survival, *n* = 132,531		Death, *n* = 4530		*P* value
	Missing	Value	Missing	Value	
Burn type, top 5, n (%)	0		0		< 0.001*
Fire/flame		32,519 (24.5)		2150 (47.5)	
Scald		29,953 (22.6)		215 (4.7)	
Contact with hot object		7737 (5.8)		52 (1.1)	
Electrical		2739 (2.1)		62 (1.4)	
Chemical		1747 (1.3)		12 (0.3)	
TBSA, % (mean ± SD)	0	8.0 ± 10.4	0	43.2 ± 29.4	< 0.001*
TBSA, full-thickness, % (mean ± SD)	25,071	2.8 ± 7.8	269	31.6 ± 30.9	< 0.001*
Inhalation injury, n (%)	31,418	5786 (5.7)	880	1682 (46.1)	< 0.001*
Intubation, n (%)	0	12,956 (9.8)	0	3664 (80.9)	< 0.001*
Ventilator, days (mean ± SD)	17,263	1.8 ± 10.1	402	14.1 ± 25.4	< 0.001*
ICU stay, days (mean ± SD)	24,375	4.1 ± 12.9	539	15.6 ± 26.5	< 0.001*
Hospitalization, days (mean ± SD)	652	9.4 ± 20.7	16	18.0 ± 31.2	< 0.001*
Trips to OpR (mean ± SD)	31,347	1.0 ± 2.8	1088	2.4 ± 5.6	< 0.001*
Operative/invasive procedures (mean ± SD)	31,347	1.7 ± 4.6	1088	4.0 ± 8.7	< 0.001*
Hospital charges, in 1000 US Dollar (mean ± SD)	83,078	79 ± 235	2791	264 ± 471	< 0.001*

*Significant with *p* < 0.05

*ICU* intensive care unit, *OpR* operating room, *SD* standard deviation, *TBSA* total body surface area, *US* United States

Variables, such as TBSA, inhalation injury, ICU stay, and the need for surgery, show how much more severe the injuries were in those patients who ultimately died after their burns

Overall, patients who died following their burn injury were more likely to suffer complications (31.9%) than those who survived their trauma (13.7%, *p* < 0.001). In our study group, these were most likely respiratory failure (12.8%), septic disorder (10.0%), renal failure (9.9%), and pneumonia (9.8%). The three most frequent causes of death reported for our 4530 deceased patients were multi-organ failure (25.6%), burn shock (16.0%), and cardiovascular shock (11.0%) in (Table 3).

**Table 3:** Morbidity and mortality of the burn patients

Variable	Survival, *n* = 132,531		Death, *n* = 4530		*P* value
	Missing	Value	Missing	Value	
Complication, any, n (%)	N/A	18,140 (13.7)	N/A	1445 (31.9)	< 0.001*
Stroke		185 (0.1)		29 (0.6)	< 0.001*
DVT		382 (0.3)		33 (0.7)	< 0.001*
ARDS		320 (0.2)		186 (4.1)	< 0.001*
Respiratory failure		1491 (1.1)		582 (12.8)	< 0.001*
Renal failure		540 (0.4)		448 (9.9)	< 0.001*
Arrhythmia		638 (0.5)		139 (3.1)	< 0.001*
Pneumonia		2155 (1.6)		443 (9.8)	< 0.001*
Urinary tract infection		1299 (1.0)		77 (1.7)	< 0.001*
Septic disorder		1548 (1.2)		451 (10.0)	< 0.001*
Wound infection		4197 (3.2)		197 (4.3)	< 0.001*
Decubitus ulcer		319 (0.2)		38 (0.8)	< 0.001*
Cause of death, top 3, n (%)	–	–	924		
Multi-organ system failure				924 (25.6)	–
Burn shock				578 (16.0)	–
Cardiovascular failure				396 (11.0)	–

*Significant with *p* < 0.05

*ARDS* acute respiratory distress syndrome, *DVT* deep venous thrombosis, *N/A* not available

It showed the patients in the deceased group suffered many more complications after their burn trauma

Multivariable analysis revealed that numerous items were independent risk factors for lethal outcomes of burns; older age (OR = 1.07) and increasing TBSA (OR = 1.09) were continuous variables related to higher mortality (*p* < 0.001). Comorbidities in general did not necessarily correlate to death after a burn injury (OR = 0.82, *p* = 0.084), but diabetes (OR = 1.24, *p* = 0.040) individually was directly linked to a higher risk of mortality. In spite of a smaller ratio of women in our study, female gender independently correlated with death due to burns (OR = 1.28, *p* < 0.001). Nevertheless, the strongest factors contributing to a lethal outcome were lack of insurance (OR = 1.84), occurrence of complications (OR = 4.09), necessity for operative or invasive interventions (OR = 2.60), and the presence of an inhalation injury (OR = 3.84). These four variables were all statistically significant in their impact (*p* < 0.001, Table 4).

**Table 4:** Multivariable regression analysis of mortality as outcome

Variable	Adjusted odds ratio	95% confidence interval	*P* value
Age^+^	1.07	1.06–1.07	< 0.001*
Female	1.28	1.17–1.41	< 0.001*
Race, African-American	1.34	1.19–1.55	< 0.001*
Uninsured	1.84	1.38–2.46	< 0.001*
Comorbidity, any	0.82	0.65–1.03	0.084
Smoker	1.21	0.97–1.50	0.086
Diabetes	1.24	1.01–1.53	0.040*
Hypertension	0.92	0.78–1.07	0.261
Obesity	0.96	0.67–1.39	0.846
Complication, any	4.09	3.27–5.12	< 0.001*
Respiratory failure	1.97	1.65–2.35	< 0.001*
Renal failure	4.87	3.89–6.09	< 0.001*
Septic disorder	1.20	0.98–1.47	0.076
Inhalation injury	3.84	3.38–4.36	< 0.001*
TBSA^+^	1.09	1.09–1.09	< 0.001*
Operative/invasive procedures	2.60	2.20–3.08	< 0.001*

*Significant with *p* < 0.05

^+^Continuous variable

*TBSA* total body surface area

**Fig. 4:**
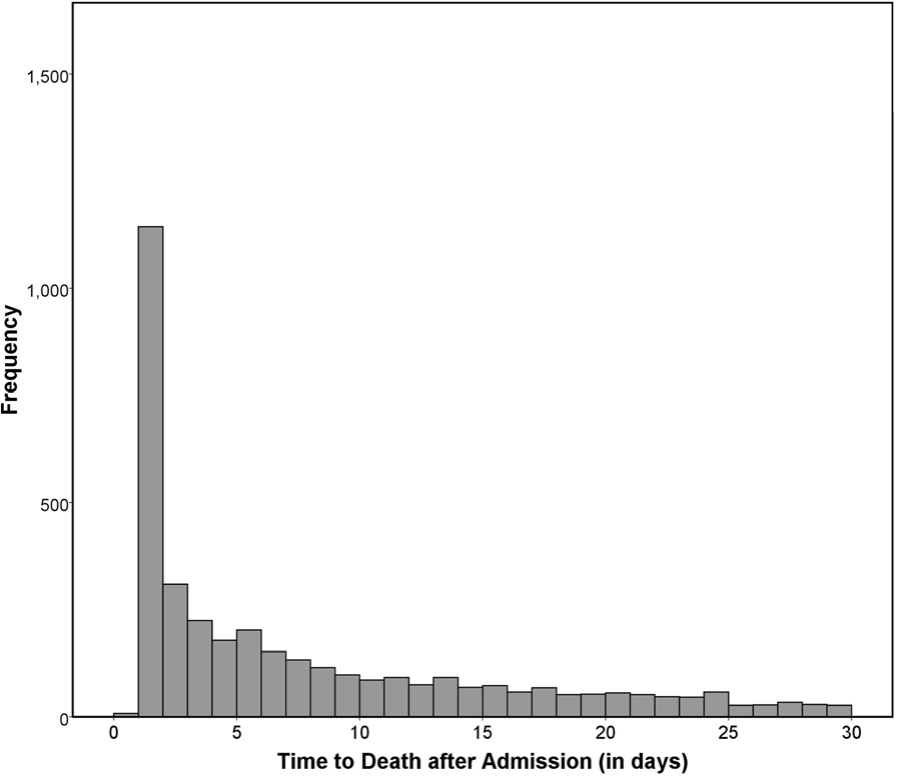
Frequency of death based on the time after admission. This figure makes it clearly evident that the majority of burn patients typically pass away within the first few days after arrival at the burn center. The graph only shows post-admission days 0–30 while the first bar (day 0) corresponds with “dead on arrival”

In the deceased group, 25.5% patients passed away by post-admission day 1, 37.3% by day 3, and 52.0% by day 7 (Fig. 4). Of those 52,052 patients that required hospitalization of 7 days or longer, 4.4% ultimately passed away. This number increased to 9.3% for patients with a minimum hospitalization/survival of 30 days (*n* = 9304) and to 11.6% with a hospitalization of 90 days or longer (*n* = 1166). Our subgroup regression analysis revealed that older patients (OR = 1.01, *p* = 0.010), those with larger burned TBSA (OR = 1.02, *p* < 0.001), with inhalation injury (OR = 1.39, *p* = 0.033), and particularly patients without health insurance (OR = 2.14, *p* = 0.002) were more likely to pass away early within 7 days rather than later. On the other hand, the patients that deceased more than 7 days after admission were more likely (*p* < 0.001) to develop complications and undergo operative procedures (Table 5).

**Table 5:** Multivariable regression analysis for early death (≤ 7 days) vs. late death (> 7 days)

Variable	Adjusted odds ratio	95% confidence interval	*P* value
Age^+^	1.01	1.00–1.01	0.010*
Female	0.93	0.79–1.09	0.364
Race, African-American	0.52	0.16–1,65	0.264
Uninsured	2.14	1.33–3.43	0.002*
Comorbidity, any	0.85	0.67–1.08	0.177
Smoker	0.87	0.60–1.26	0.455
Diabetes	1.01	0.71–1.45	0.938
Hypertension	1.15	0.88–1.50	0.295
Obesity	0.75	0.41–1.37	0.346
Complication, any	0.41	0.32–0.53	< 0.001*
Respiratory failure	1.18	0.90–1.55	0.227
Renal failure	0.58	0.42–0.79	0.001*
Septic disorder	0.17	0.12–0.25	< 0.001*
Inhalation injury	1.39	1.03–1.87	0.033*
TBSA^+^	1.02	1.02–1.03	< 0.001*
Operative/invasive procedures	0.19	0.14–0.26	< 0.001*

Early death: *n* = 2356; late death: *n* = 2174

*Significant with *p* < 0.05

^+^Continuous variable

*TBSA* total body surface area

## Discussion

The current database literature examining the risk factors for morbidity and mortality after burns typically focuses on specific variables, such as age, gender, or socioeconomic status [6, 10, 11]. In these cases, the true risk amplification, which is illustrated by the OR and respective 95% CI, may be distorted if the multivariable analysis is not broad enough to include variables, such as comorbidities and in-hospital complications. Other authors may use datasets that are not up-to-date any longer or contain invalid data and thus mandate reevaluation [7, 11, 12]. Additionally, older versions of the NBR were indeed prone to missing information [2]. Single-center studies, on the other hand, usually face statistical limitations of low case numbers with an enrolled patient population that may not be representative for every clinical setting in the US [13–18]. The purpose of this report was to identify the strongest risk factors for mortality in burn patients using a large multi-institutional database as guidance for better patient management and outcome prognosis.

In this study, patients in the death cohort were almost twice as old, more likely a female, Caucasian or African-American, and also had significantly increased rates of comorbidities and health-hazardous behavior such as smoking or alcohol use. Interestingly, these comorbidities—with the exception of diabetes—did not statistically contribute to the patients’ mortality. The NBR collects a wide variety of comorbidities and includes nonspecific categories, such as “steroid use,” “psychiatric illness,” and even “others,” which may attenuate the significance of comorbidities in general. Pompermaier et al. also argued that comorbidities play an inferior role in comparison to age in burn mortality models [19]. On the other hand, Knowlin et al. demonstrated in a single-center study that comorbidities are in fact associated with increased mortality after burns [20]. In our outcome analysis, other clinical factors were more predominant. Discrepancies in immune response have been suggested as an explanation for the increased mortality of females [21]. Variables, such as TBSA (mean 43.2% in death cohort), inhalation injury (46.1%), any form of in-hospital complications (31.9%), and subsequent operative procedures (mean 4.0), were more decisive for our deceased patients and reached significance in the multivariable analysis. Given the fairly low rates of pre-existing comorbidities (respiratory disease 6.4%), the majority of post-operative complications in deceased patients, such as respiratory failure, pneumonia, and septic failure, can be confidently related to the burn trauma itself. In addition, we demonstrated through multivariable regression that a lack of health insurance comes with a similar risk (OR = 1.84, *p* < 0.001) as a respiratory failure (OR = 1.97, *p* < 0.001). Interestingly, uninsured patients had a similar total TBSA (9.3% vs. 9.1%, *p* = 0.054) and full-thickness TBSA (3.7% vs. 3.9%, *p* = 0.053) on univariate analysis compared to insured patients. Therefore, insurance or socioeconomic status does not necessary put someone at risk for a more severe burn injury, but it does contribute to fatal outcomes. In addition, the lack of health insurance is a significant risk factor for early death within 7 days (Table 5). In critical and especially life-threatening injuries such as burns, healthcare providers in the US are required to provide maximum treatment regardless of economic considerations. Nevertheless, lack of insurance appeared as a significant risk factor [22]. In 2010, the Affordable Care Act was passed giving more people access to health insurance and reducing their general mortality rates [23]. We are confident that these positive trends will be similar in the burn population. Additionally, African-American race was a significant independent factor contributing to mortality risk although African-Americans had higher uninsured status (15.7%) compared to Hispanics (14.9%) or Caucasians (12.5%). Patients who did not survive their burn injuries required longer hospitalization times, including intensive care unit (ICU) stays, and suffered more complications, which often had to be addressed with invasive procedures and trips to the operating room. The interaction of these aggravating circumstances increased the final inpatient costs.

Strassle et al., similar to our results, reported from a single burn center where increasing age and TBSA, as well as inhalation injury, were identified as hazards related to mortality. However, they could not reach significance for the following variables, such as gender or ethnicity. Their graphs showed how prolonged hospitalization increased overall mortality similar to our univariate analysis [18]. O’Keefe and colleagues faced connatural statistical limitations in their single-center design where numerous calculations did not reach significance [17].

Studies using the NBR as primary data source generally achieved results analog to ours. Age, gender, race, TBSA, and an inhalation injury were significant for mortality in Bedri et al.’s regression model, yet the authors did not include the occurrence of complications or the need for operative procedures. In their study, operative procedures were a primary outcome rather than an independent variable as used in our report. Furthermore, the authors did not exclude cases with missing death/survival outcome (variable “dead” in NBR) before performing any statistical analysis regarding mortality [6]. Perhaps, it was for this reason why they reported concomitant comorbidities as risk factors for death. On the other hand, in our report, only diabetes was determined as a significant contributor to mortality.

Some previously mentioned variables were detected as significant in even an older version of the NBR database yet their risk amplification differed [24]. Taylor et al., on the other hand, merely focused on age and TBSA as variables correlating with death after burn injury. They fittingly concluded that pediatric patients should be evaluated separately from adults and the elderly since the importance of risk factors varies by cohort [10]. Age, TBSA, inhalation disorder, comorbidities, and lack of available beds were also mortality risks in a large British multi-institutional analysis. These findings indicate that the burdens of burn injuries are internationally universal regardless of the healthcare system. [25] The Baux [26] and the FLAMES (Fatality by Longevity, acute physiology and chronic health evaluation II (APACHE II) score, Measured Extent of Burn, and Sex) score [15] are prognostic factors that are often encountered in burn research. However, they are created by compiling two or more measures, such as age, gender, TBSA, or the APACHE II score, into one composite variable as a form of outcome estimate and may yet again lack the input of other risk factors.

Inhalation injury, burn complications, operative procedures, and lack of insurance were identified as major risk factors in this study. Patients of African-American ethnicity were at highest risk of death than any other subgroup. Age and TBSA illustrated predictors for mortality on a continuous scale. Knowing that the majority of patients who do not recover from their trauma pass away within the first 7 days [27], identifying most of these risk factors early after admission and knowing their magnitude is crucial to provide optimal care. In the same vein, complications need to be addressed as soon as they develop. Physicians and staff should focus on factors that can be modified, such as treatment of inhalation injury or the prevention of complications. Meanwhile, other factors, such as gender, age, or ethnicity, are inherent to an individual patient and simply need to be kept at the back of one’s mind. In cases where a highly adverse prognosis is established, withdrawal of life support may represent a sensible option for these patients [28]. Furthermore, after the initially critical period after burn injuries when most deaths occurred (Fig. 4), patients had increasing mortality rates with 7, 30, and 90 days of hospitalization—4.4, 9.3, and 11.6%, respectively. Some of the results presented in this article play a role in the current development of the Burn Quality Improvement Program (BQIP) [29], which may ultimately produce a calculator to estimate realistic outcomes of burned patients using data collected in the NBR. We employed a large multi-center database in a detailed multivariable analysis to assess the individual impact of each perceivable risk factor. The variety of results in the literature shows that regression models need to be carefully designed for accurate hazard estimates.

Our study is not without limitations. The NBR is an administrative database that is tended to by the institutions and their study staff who upload the patient details that depend on accurate reporting and are subject to individual errors. The countless participants included in our study come from a vast variety of institutions. Certain US states do not operate burn centers or none that reported to the NBR within our study period (Fig. 2). Our results may therefore vary between hospitals. Furthermore, most variables have experienced modifications over the recent years and may include portions with missing data, which we had to disregard or remove from our analyses as described in this article’s methodology. Comorbidities and complications reported in this article originate from separate files, which had to be carefully restructured and merged with the main dataset containing the majority of variables. Furthermore, information about prior treatment at other hospitals is not provided for patients who are transferred to an NBR burn center. Although the NBR provides the cause of death for most cases, it does not report how often withdrawal of treatment occurred. Due to the nature of the database, we also cannot evaluate various surgical techniques used in the treatment of our patients who had an average of 1.7 ± 4.6 (survival group) and 4.0 ± 8.7 (death group) procedures performed during their hospitalization. The odds ratios of our regression analysis may fluctuate between different patients who may present with different types of burns and different Abbreviated Burn Severity Index (ABSI) scores [30]. The risks identified in this study originate from thousands of cases provided by the NBR and are average figures of US burn centers.

As this is a retrospective study, all significant findings have associative and not causative character.

## Conclusion

This study is one of the first that analyzed various risk factors and their individual impact on death after burn injuries on a large multi-institutional scale. The diagnosis of inhalation injury, the occurrence of inpatient complications, the need for operative or invasive procedures, and lack of health insurance were determined as leading contributors to mortality. Age and burned TBSA continuously increased the probability of death. Knowing that the majority of critical burn patients (52.0%) passed away within the first 7 days of admission, physicians and staff in burn centers should be aware of these risk factors. Additional research is warranted to help understand why certain patient characteristics (uninsured status, female gender) are such significant contributors for mortality after burns.

## Abbreviations

ABA: American Burn Association
ABSI: Abbreviated Burn Severity Index
APACHE II: Acute physiology and chronic health evaluation II
ARDS: Acute respiratory distress syndrome
BQIP: Burn Quality Improvement Program
CHF: Chronic heart failure
CI: Confidence interval
CVA: Cerebrovascular accident
DVT: Deep venous thrombosis
FLAMES: Fatality by Longevity, APACHE II score, Measured Extent of Burn, and Sex
ICU: Intensive care unit
MI: Myocardial infarction
NBR: National Burn Repository
N/A: Not available
OpR: Operating room
OR: Odds ratio
SD: Standard deviation
TBSA: Total body surface area
US: United States
